# Is There an Association between Temporomandibular Disorders and Articular Eminence Inclination? A Systematic Review

**DOI:** 10.3390/diagnostics11010029

**Published:** 2020-12-26

**Authors:** Xiao-Chuan Fan, Diwakar Singh, Lin-Sha Ma, Eva Piehslinger, Xiao-Feng Huang, Xiaohui Rausch-Fan

**Affiliations:** 1 Department of Stomatology, Beijing Friendship Hospital, Capital Medical University, Beijing 100050, China; foxtail_09@hotmail.com (X.-C.F.); malinthe@yeah.net (L.-S.M.); 2 Division of Conservative Dentistry and Periodontology, School of Dentistry, Medical University of Vienna, Vienna 1090, Austria; dentistdiwakarsingh@gmail.com; 3 Division of Prosthodontics, School of Dentistry, Medical University of Vienna, Vienna 1090, Austria; eva.piehslinger@meduniwien.ac.at

**Keywords:** temporomandibular disorders, inclination of articular eminence, temporomandibular joint, glenoid fossa

## Abstract

(1) Background: In order to determine the correlation between the inclination of articular eminence (AEI) and the development of temporomandibular disorders (TMDs), a systematic review was performed. (2) Methods: A systematic literature research was conducted between 1946 and January 2020, based on the following electronic databases: PubMed, Cochrane Library, Embase, Medline, Scope, SciELO, and Lilacs. Observational studies, analytical case-control studies, and cohort studies written in English were identified. The articles were selected and analyzed by two authors independently. The PICO format was used to analyze the studies and the Newcastle-Ottawa Scale (NOS) was used to verify the quality of the evidence. (3) Results: Sixteen articles were included in this review, ten case-control studies and six cohort studies. Eight articles (50%) established a positive relation between AEI and TMDs and eight (50%) did not. The scientific quality was medium-low, mainly influenced by the exposure to the risk of bias and the lack of clinical methods with adequate consistency and sensitivity on the diagnosis of TMDs. (4) Conclusions: It is controversial to establish a causal relationship between the TMDs and the AEI in the field of stomatology, due to limited and inconclusive evidence. However, it is suggested that the AEI defined by some specific methods may be associated with some special pathological stages of TMDs. High-quality prospective studies are required to draw any definitive conclusions.

## 1. Introduction

The temporomandibular joint (TMJ) is one of the most complex articular systems in human beings, which is formed by the glenoid fossa of the temporal bone (the superior component of the joint), and the mandibular condyle (the inferior component of the joint) and the two are separated by the articular disk [1,2]. The anatomy of the TMJ can provide capacity in both hinging movement and gliding movements of the mandibular within the three planes of space. The TMJ is critical to the craniomandibular system because it can achieve the mandibular functions with a dynamic balance mechanism [3]. Over the years, numerous studies have focused on the relation of the change of anatomical and physiological characteristics to stomatognathic dysfunctions [4], especially in cases of joint disorders [5].

Temporomandibular disorders (TMDs) are one of the most prevalent pathologies, which are defined as a comprehensive term of disorders affecting the TMJ, the muscles involved in mastication and/or the related structures [6]. Epidemiological studies of non-patient adult populations have shown that about 40–75% of patients have at least one sign of joint dysfunction, such as joint clicking, abnormal movement, and 33% of them have joint or facial pain [6]. Although the prevalence of TMDs in the population has attracted more attention from clinicians and researchers over the years, the etiology of TMDs is still poorly understood and remains to be elucidated [7,8].

Numerous factors that contribute to the development of TMDs have been proposed, such as traumatic injuries, occlusal disharmony, psychological factors, luxation of the joints, loss of posterior teeth, spine and postural alterations, and muscle hyperactivity [9,10,11,12]. Beside these factors, the features of the anatomic structure of the TMJ are also considered to be a local factor involved in the development of TMDs. During functional movements of the mandibular, the condylar process slides along the posterior slope of the articular eminence. A change of inclination of articular eminence might result in biomechanical variations of the TMJ because its characteristics determine the trajectory of functional movement [13]. Therefore, we speculate that articular eminence steepness and mandibular fossa morphology may have some connections with certain diseases that induce TMJ.

The relationship between the TMDs with the articular eminence inclination (AEI) has been investigated by previous studies. However, the associations between these two indicators have been found to be inconsistent and definitive conclusions cannot be drawn [14,15,16,17,18,19,20]. On the basis of these premises, a well-designed systematic review is needed to clarify this opening question. This study attempts to systematically review the literature to find out the correlation between the inclination of articular eminence and the development of TMDs, analyzing the quality of the methodological soundness of previous studies.

## 2. Materials and Methods

In order to answer the research question about the relationship between the AEI and TMDs, a systematic search of the medical literature was performed on 17 June 2019 and updated on 27 January 2020. Databases used were as follows: PubMed, Cochrane Library, Embase, Medline, Scope, SciELO, and Lilacs.

### 2.1. Protocol

This systematic review was reported following the guidelines of the Preferred Reporting Items for Systematic Reviews and Meta-Analysis (PRISMA) checklist [21].

### 2.2. Types of Studies

Observational studies, analytical case-control studies, or cohort studies aimed to determine the relationship of the inclination of articular eminence to the occurrence of TMDs.

### 2.3. Language Studies

The search was limited to articles in peer-reviewed journals and written in the English language.

### 2.4. Types of Participants

The studies selected for this review included subjects of both genders without the limitation of age.

### 2.5. Intervention Type

Studies without intervention in order to correlate AEI and TMDs.

### 2.6. Type of Results

The primary outcome was to determine the relationship between AEI and TMDs.

The secondary outcome was to determine AEI and the morphology of glenoid fossa related to the different pathological stages of TMDs.

### 2.7. Data Collection

For TMDs, the data were collected from studies that showed the diagnosis of TMDs with a clear reference to the concept and diagnosis of temporomandibular pathology in any method without limitation. Diagnostic criteria for TMD was based on research diagnostic criteria for temporomandibular disorders (RDC/TMD), diagnostic criteria for temporomandibular disorders (DC/TMD) [22,23], evaluation according to the American Academy of Orofacial Pain (AAOP) guide [6], radiology studies (including magnetic resonance imaging (MRI), computed tomography (CT), cone-beam computed tomography (CBCT), sagittal corrected tomography, arthrography, and other methods), Helkimo index, surveys’ studies, and/or clinical examination based on signs and symptoms with reference to TMD and others.

For AEI, the data were collected from studies that showed a clear method for measuring the AEI in degrees. The AEI is defined as the angle between the articular eminence and the Frankfort horizontal (FH) plane or any other horizontal reference plane, such as the palatal plane, the occlusion plane, the anterior nasal spine to the posterior nasal plane (ANS-PNS), and other defined reference planes. Data were collected based on MRI, CT, CBCT, tomography, dry skulls, autopsy, and other methods.

### 2.8. Databases Used

1. PubMed database (article types, clinical trials, randomized controlled trials, controlled clinical trials; language, English; publication dates, 1 January 1966 to 27 January 2020);
2. Cochrane Library (database, Trials; publication dates, 1966 to 2020);
3. Embase (publication dates, 1974 to 2020)
4. Medline (publication dates, 1946 to 2020)
5. Scope (document type, article; language, English; publication dates, 1970 to 2020)
6. SciELO (publication dates, to 2020)
7. Lilacs (publication dates, to 2020).

### 2.9. Search Strategy

A systematic search of the computerized database was performed to identify and select the potentially eligible literature that examined the association between AEI and TMDs for this systematic review. The semantic field related to the term “TMDs” (temporomandibular disorders, TMJ dysfunction, disk displacement, muscular pain, clicking) was crossed search with the semantic field related to the term “AEI” (glenoid fossa, posterior slope, articular eminence). For details regarding the specific search terms and combinations, see Table 1.

### 2.10. Study Selection

For article selection or first approach, all potentially eligible articles were listed by title and abstract and evaluated by two researchers independently (X-C.F. and D.S.). Then, the full text of articles, which may meet the inclusion criteria based on the first stage of selection, was assessed independently by the same two researchers (X-C.F. and D.S.). When no agreement was found during the first and second stage of selection, the data was discussed with a third researcher (X.R.F.), to reach final decision for including it or not. When the full-text version of the study was not directly available, the paper was requested from the corresponding author by email. Articles that met all inclusion and exclusion criteria were selected in the review for the final analysis. The reasons for the exclusion of the articles were recorded in an adjacent column and presented in the results (Table 2).

### 2.11. Extracting Data from the Studies

The methodological features of the selected articles were assessed according to a format, the PICO criteria, which enabled a structured summary of the analyzed articles in relation to four main issues, namely, population, intervention, comparison, and outcome. For each article, we defined the following analysis variables in detail: population (sample size, distribution by gender, mean age, and age range); intervention (type of method used for the diagnosis of TMDs, main variables to compare, statistical analysis); comparison (assessed the presence of any comparison groups); outcomes (the answer to the hypothesis, the presence of causal relationship between AEI and TMD). Some studies investigating more items were reported in two or more groups of correlation.

### 2.12. Quality Assessment

Critical appraisal of studies included in the review was determined by the Newcastle-Ottawa Scale (NOS), which was used to assess the quality of case-control and cohort studies [39]. To determine the quality of case-control studies, there were three categories with a level of evidence score ranging from 0 to 9 points as follows: (1) selection (four points), (2) comparability (two points), and (3) exposure (three points). For cohort studies, there were also three categories assigning a score ranging from 0 to 9 points as follows: (1) selection (four points), (2) comparability (two points), and (3) outcome (three points).

The quality was determined by the same two researchers (X-C.F. and D.S.) in charge of the search, where the highest quality achieved was obtained by those items that were assigned a maximum score of 9.

## 3. Results

**Figure 1:**
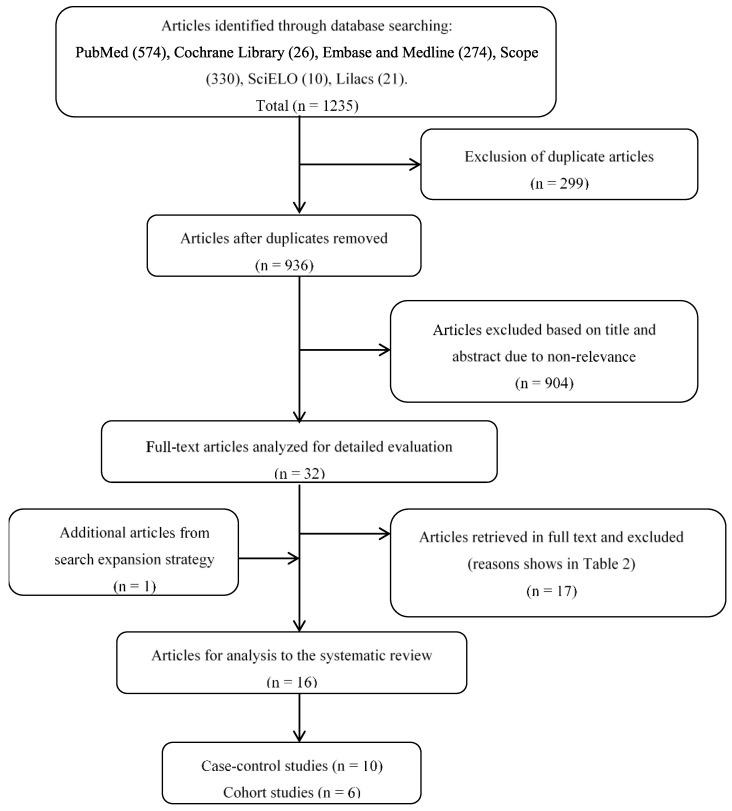
Search method, identification, selection, and inclusion of articles.

In total, 1235 potentially eligible articles were examined in the first approach in the seven databases used (Table 1). However, 299 of these articles were excluded due to duplication. On the basis of the title and abstract of the remaining 936 studies, 904 of them were eliminated due to their lack of relevance. Of the 32 articles left, after reading the full text, a consensus decision was to eliminate 17 articles that did not fulfill the inclusion criteria for this systematic review. Table 2 reveals the list of excluded studies including the reason for exclusion. Search expansion strategies allowed including one additional paper, thus, accounting for a total of 16 studies that were analyzed in the review and prepared according to the PICO criteria (Table 3, Table 4 and Table 5). Figure 1 summarizes the search strategy and results described.

### 3.1. Characteristics of Studies

In all, 16 articles were included in this systematic review. ten case-control studies and six cohort studies were identified. According to the radiological methods, four are MRI studies, seven are CT or CBCT studies, and the other five articles used two-dimensional (2D) radiographs (sagittal corrected tomography or lateral oblique transcranial radiographs). Among these articles, two of them used both three-dimensional (3D) (CT or MRI) and two-dimensional (2D) radiographs (lateral cephalogram or laminography) [45,48] (Table 3, Table 4 and Table 5). We selected these two articles for a single group, because the other radiological methods were not used to evaluate the AEI after reading of the full text.

### 3.2. Characteristics of Participants

The age range of patients was between 14 and 88 years; the average age was from 19.1 ± 4.7 to 40.92 years. Three studies did not specify the age of the participants clearly [3,41,48]. In relation to gender, four studies included only females in their sample [19,45,46,47].

### 3.3. Quality Assessment

Among the total 16 articles selected in this review, eight studies presented correlations between TMDs and the AEI, however, the other eight studies did not find any correlations between the TMDs and the AEI. None of the included studies obtained the highest score based on NOS. The range of scores was between two and six (Table 6).

## 4. Discussion

A review of literature can help us gain knowledge more effectively, however, it is necessary to carefully analyze the quality, to avoid erroneous conclusions from their results. The objective of this systematic review was to select and analyze the studies that verify the correlation between the inclination of articular eminence and specific TMD signs and symptoms, presenting real applicability to clinical practice.

According to the inclusion and exclusion criteria of this review, the search was conducted with the limitation of peer-reviewed English language papers, although this strategy may lead to the possibility that some publications in other languages and/or publications included in databases were unjustly excluded. However, it is a way to improve the methodological rigor and the conclusion drawn to a certain extent. Case reports and reviews are also excluded, as they do not have uniform standards that could increase the risk of bias.

From a methodological point of view, all the articles selected in this systematic review were retrospective observational studies with or without control groups verifying the correlation between AEI and TMDs. The scientific quality of evidence of the analyzed studies included in the present review was medium-low, mainly influenced by the exposure to the risk of bias and the lack of clinical methods with adequate consistency and sensitivity used for the diagnosis of TMDs. One of the methods created with the purpose of clinical and epidemiological research used for the diagnosis of evidence-based TMDs is the RDC/TMD, and the other method is the DC/TMD, which results in an evidence-based system with greater validity for clinical use [23]. The RDC/TMD criteria and the DC/TMD criteria are emphasized as the international standard for examination of patients, which have existed since 1992 or 2014. Therefore, the qualities of all studies before that time are evaluated as weak. All of the selected articles in this systematic review, except for one (Panmekiate, 1991 [49]), were published after 1992. The types of method used for the diagnosis of TMDs of the selected articles were shown in the ”intervention” part of Table 3. However, only four studies, included in the review, diagnosed TMDs and classified samples according to RDC/TMD criteria [19,40,43,44], two studies diagnosed TMDs based on Helkimo index [14,18], and others diagnosed TMDs only by clinical sign and symptoms or then further confirmed by MRI or arthrography. That means the inclusion criteria of the papers are not consistent. The lack of introduction of uniform diagnostic criteria, such as RDC/TMD or DC/TMD defining the different categories of TMDs, decreases the level of consistency, resulting in a low quality of studies, and therefore comparisons between different studies could not be established. Without consistency, may imply that the observed correlations between two variables appeared because of chance or error [50]. Furthermore, TMDs are considered to be a heterogeneous group of different diseases involving the craniomandibular system, other than a single pathology [51]. It is difficult to control for all of the other variables when evaluating the relative importance of single risk factors for disorders with a multifactorial etiology [52,53]. Some studies that still seem to continue to use ”TMDs” as a collective term of all TMD signs and symptoms during the clinical examinations, pooled them in a unique dependent variable in the statistical analysis and the results [3,43,44]. Nevertheless, the evaluation of the multifactorial complex pathologies, such as TMDs, should use multivariate statistical analyses, as univariate models may overestimate some resulting associations and possibly produce misleading conclusions [54,55]. This could be shown from the study of Rabelo KA et al. [17], who found an important correlation among the type of disk displacement of the AEI (*p* < 0.001), but there was no statistical correlation between the presence and absence of disk displacement of AEI measurements (*p* > 0.05). Similarly, the AEI was steeper in the no condyle bone change group than in those of the bilateral condylar bone change (centre section *p* < 0.05, lateral section *p* < 0.01). However, these differences were only seen in the joints with osteophyte (all three sections *p* < 0.05) but not with erosion (all three sections *p* > 0.05), based on the study of Yamada K et al. [46].

Many radiographic methods have been selected to measure the AEI in previous studies. In the early days, conventional radiographs, such as tomography or arthrography, were used for diagnosing the morphology of TMJ, but these modalities proved to have certain limitations [1] and were replaced by helical CT, which evaluates osseous components in 3D without superimposition or distortion. The CBCT, which has high dimensional accuracy in measuring maxillofacial structures including TMJ, is considered to be one of the preferred ways to evaluate bone structure in the stomatological area [3,14,18]. Nowadays, CBCT was selected rather than helical CT because of lower radiation dose, better spatial resolution, shorter scanning time, and more cost effective [56]. The MRI also allows a tridimensional analysis of the TMJ, and this technology can provide hard tissue and also soft tissue imaging, such as articular disk, related muscles, and ligaments. It has already been considered to be the gold standard imaging method for the diagnosis of internal derangement and the disk displacements with or without reduction. The radiographic methods are very important factors for angular and linear measurements as it influences the results. The articles included in this systematic review involve five imaging methods, from two-dimensional methods to three-dimensional methods. Using 3D imaging, the steepness of the eminence may be influenced by the location of the image (more laterally, centrally, more medially), whereas the 2D images show a summarization of the whole articular eminence as a three-dimensional structure. It is hard for us to establish comparisons of the values of AEI between different studies with different imaging methods because the consistencies of them still need more studies to support.

**Figure 2:**
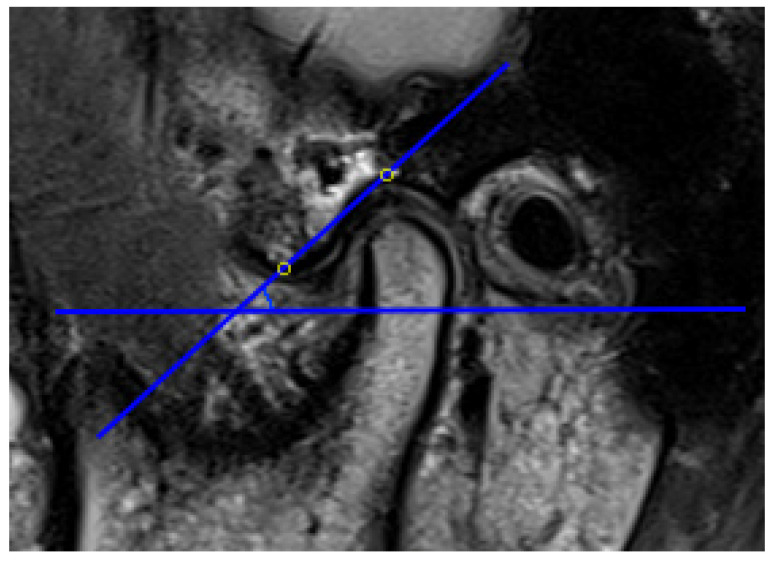
Representative images of “top-roof line” method, the articular eminence inclination (AEI) defined as the angle between the line connecting the crest point of the articular eminence and the roof of the mandibular fossa and the horizontal reference plane.

**Figure 3:**
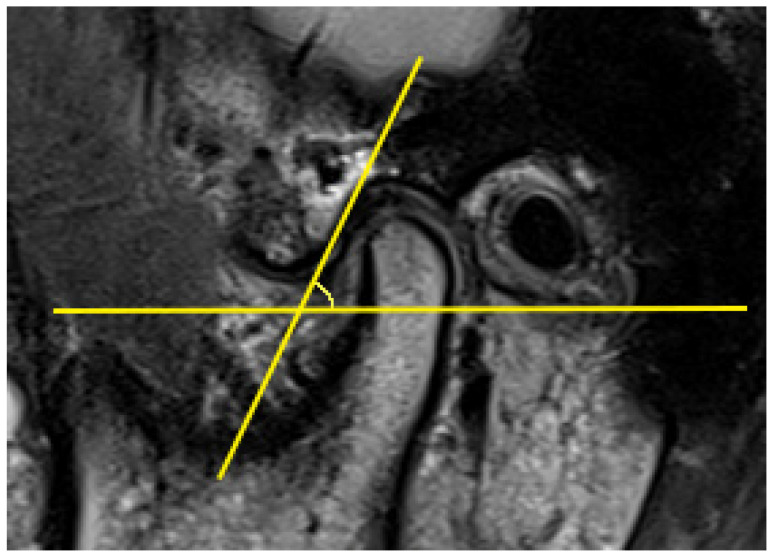
Representative images of the “best-fit line” method, the articular eminence inclination (AEI) defined as the angle between the tangent line drawn to the posterior slope of the articular eminence and the horizontal reference plane.

The AEI is defined as the angle formed by one of the lines that passes through the articular eminence and the horizontal reference plane [57]. In previous articles, two main methods have been described for evaluating the AEI, i.e., the “top-roof line” method and the “best-fit line” method, which are reliable and have already been used in studies. The “top-roof line” is obtained by connecting the crest point of the articular eminence and the roof of the mandibular fossa (Figure 2). The angle between the “top-roof line” and the horizontal reference plane is related to the height of articular eminence, which focuses on the localization of the tubercle in relation to the mandibular fossa and depicts the morphology of articular eminence better. The “best-fit line” method was defined as the angle between the tangent line drawn to the posterior slope of the articular eminence and the horizontal reference plane, which is directly related to the movement direction of the condyle-disk complex and reflects the actual condylar path (Figure 3) [26,30,31,41,57,58]. Five of 16 articles selected in this systematic review used the “top-roof line” method [14,17,18,43,49]; eight articles used the “best-fit line” method [19,20,40,41,42,45,46,48]; two articles used both the “top-roof line” method and the “top-roof line” method to evaluate the AEI [3,47], and the other article used the angle between tangent line from the uppermost point of the glenoid fossa and the true horizontal line as AEI [44]. Although the three mentioned methods all represent the inclination of the articulator eminence, the features they focus on are different. Therefore, they should be considered separately.

The horizontal reference plane is the other important factor affecting the AEI, which determines the degree of the angle directly. At the stage of the literature selection, the reference planes used were not limited, which can be FH plane, palatal plane, occlusion plane, and other defined reference planes. Except for three studies (one study used the true horizontal line [44], one study used the line tangent to the anterior and posterior articular eminences [19], one study used the line tangent to the curve of articular eminence and the point of squamotympanic fissure [47]), and the remaining 13 studies included in this review all used the FH plane as horizontal reference planes. It has been generally recognized as an important reference plane and has proved to be of great value in cephalometric analysis and the three dimensions measurement since the Frankfurt agreement concluded in Germany in 1884, which is defined by a line drawn from the lowest point on the inferior orbital margin (Or) to the most superior point of the outline of the external auditory meatus (Po) [59,60]. A stable and comparable horizontal reference plane is very essential, and the FH plane seems to be a relatively ideal reference plane for evaluating AEI because the landmarks of the FH plane are independent of TMJ structure, which are not affected by the changes of mandibular fossa and articular eminence. However, although FH is well defined, the external auditory meatus changes its shape looking on a more lateral, central, or medial slice of MRI, CT, or CBCT, which may also influence the steepness of the articular eminence.

Another important confounding factor in the analysis of the correlation between TMDs and AEI may be represented by the selection of the samples. Some of the studies were based on orthodontic patients [45,46], who may be alerted to the potential role of malocclusion as a risk factor of TMD. The control group or the asymptomatic volunteers of some studies was selected from the dental students [42], who can be aware of the risk factors of the TMDs and avoid them. In such cases, the samples selected may hardly represent the general population. The genders of the samples included also have such a problem. The groups of symptomatic patients in most studies included in this systematic review contain more female than male, which has a significant difference in gender distribution from the general population [3,14,17,18,20,42,45,48,49]. However, TMDs affect approximately 40% to 75% of the general adult population, 80% of which seeking for TMD are females. Milano et al. reported that disk displacements of TMJ appeared considerably more often in females than in males because of altered collagen metabolism associated with joint laxity of genetic origin [61]. Peroz et al. also found that females present a greater correlation with disk displacements than males [62]. According to Warren and Fried, estrogen may influence the development and metabolism of the TMJ and associated structures (include bone, cartilage, and articular disk), and it may also influence the pain regulation mechanism [63]. The evidence in the previous articles suggested that the pathogenesis of TMDs may have a possible link with estrogen and that TMDs is more prevalent in the female. Therefore, we also included four articles containing only female subjects [19,45,46,47].

The development stage of the articulation may also influence the AEI. According to the previous studies [64,65], from newborn to infancy, the articular surface was largely flat and the articular eminence was poorly developed. From the stage of the end of the primary dentition to mixed dentition, the fossa and the articular eminence had clearly developed and completed approximately 45% of its development, but the articular eminence was still fairly flat. Around the age of 10 years old, articular eminence completed approximately 70–72% of its development. The fully developed time of the articular eminence is still controversial. From the study by Katsavrias and Dibbets [65], articular eminence was 90–94% complete by the age of 20 years. However, based on the autopsy study published in 1971 [64], tubercle and the fossa were well developed at the age of 14–15 years. This review presents a high variability in the age range of 14–88 years. A poorly developed fossa may show a flatter tendency, which may possibly produce misleading conclusions.

This systematic review retrieves and analyzes the medical literature about the relationships between the TMDs and the AEI published in seven databases over the past 74 years, 50% of the studies showed a positive correlation between the TMDs and AEI, but the evidence is not in high quality. In relation to the findings in this review, the following suggestions can be drawn:

1. The correlation between TMDs and AEI is still an unsolved issue. Definitive conclusions cannot be drawn based on the present studies.
2. Evidence-based diagnosis with TMDs was not uniform. It is suggested to use multivariate statistical analyses for the evaluation of multifactorial complex pathologies such as TMDs.
3. The insufficient number of articles considered of high methodological quality is a factor that hinders the acceptance or denial of this correlation.
4. More quality and carefully designed prospective studies are required by future researchers to determine the causal relationship between TMDs and AEI.

## 5. Conclusions

Definitive conclusions cannot be drawn based on the quality of evidence available, since the definition and clinical methods were very heterogeneous and presented a high risk of bias. The insufficient number of articles considered of high methodological quality is another factor that hinders the acceptance or denial of this correlation. However, it is suggested that the AEI defined by some specific methods may be related to some special pathological stages of TMDs to a certain extent. Well-designed prospective studies are required to draw any further definitive conclusions.

## Figures and Tables

**Table 1:** Search strategy and terms used for the search.

Database and Limits	Search Strategy and Terms		
	Semantic Fields: Temporomandibular Disorders		Semantic Fields:Articular Eminence Inclination	
PubMed (n = 574) Article types, clinical trials, randomized controlled trials, controlled clinical trials Language, English Publication dates, 01 January 1966 to 27 January 2020	Temporomandibular disorder [tiab] OR TMJ Dysfunction [tiab] OR disk displacement [tiab] OR Muscular pain [tiab] OR clicking [tiab]	AND	glenoid fossa [tiab] OR posterior slope [tiab] OR articular eminence [tiab]
Cochrane Library (n = 26) Database, trials Publication dates, 1966 to 2020	Temporomandibular disorder OR TMJ Dysfunction OR disk displacement OR Muscular pain OR clicking	AND	glenoid fossa OR posterior slope OR articular eminence
Embase and Medline (n = 274) Publication dates, Embase 1974 to 2020 and Medline 1946 to 2020	Temporomandibular disorder OR TMJ Dysfunction OR disk displacement OR Muscular pain OR clicking	AND	glenoid fossa OR posterior slope OR articular eminence
Scope (n = 330) Document type, article Language, English Publication dates, 1970 to 2020.	(TITLE-ABS-KEY (temporomandibular AND disorders) OR TITLE-ABS-KEY (tmj AND dysfunction) OR TITLE-ABS-KEY (disk AND displacement) OR TITLE-ABS-KEY (clicking) OR TITLE-ABS-KEY (muscular AND pain) AND TITLE-ABS-KEY (articular AND eminence) OR TITLE-ABS-KEY (glenoid AND fossa)) AND (LIMIT-TO (DOCTYPE, “ar”)) AND (LIMIT-TO (LANGUAGE, “English”))		
SciELO (n = 10) Publication dates, to 2020	(Temporomandibular disorder OR TMJ Dysfunction OR disk displacement OR Muscular pain OR clicking)	AND	(glenoid fossa OR articular eminence)
Lilacs (n = 21) Publication dates, to 2020	(Temporomandibular disorder OR TMJ Dysfunction OR disk displacement OR Muscular pain OR clicking)	AND	(glenoid fossa OR articular eminence)

**Table 2:** Studies retrieved in full text and excluded from the review.

First Author and Year	Reason for Exclusion
de Pontes, 2019 [24]	Morphological research
Shokri, 2019 [25]	Quantitative data of AEI is not shown
Piancino, 2020 [16]	Concerning on TMDs patients with or without condylar asymmetry
Sa, 2017 [26]	Patients with degenerative bone diseases
Rabelo, 2017 [27]	No direct relationship between fossa shape and TMDs
Türp, 2016 [28]	No direct relationship between AEI and TMDs
Su, 2014 [29]	Grouping of the glenoid fossa is not clear
İlgüy, 2014 [30]	The diagnosis of participates is not clear
Çağlayan, 2014 [31]	Same data as Sümbüllü, 2012 [3]
Learreta, 2013 [32]	Group divided based on alterations in the condylar axis
Robinson de Senna, 2009 [33]	No description of the morphology of the fossa
Hirata, 2007 [34]	Sample size is too small
Kurita, 2006 [35]	Morphological research
Tanaka, 2004 [8]	Dry skull study, no TMDs diagnosis
Pullinger, 2001 [36]	Same data as Pullinger, 2002 [19]
Kurita, 2000 [37]	Grouping is not clear
Toyama, 1999 [38]	Only the relationship between disk and fossa

**Table 3:** Summary of findings from studies of TMDs and AEI with MRI.

First Author & Year	Type of Study	Population	Intervention	Comparison (Control Group)	Outcome	Conclusions
Poluha, 2020 [40]	Case-control study	36 individuals: 12 DDWR and arthralgia (12F, m.a.: 33.58 ± 9.75), DDWR and no arthralgia (4M, 8F, m.a.: 32.58 ± 10.9), asymptomatic individuals (3M, 9F, m.a.: 29 ± 6.86)	TMDs: symptoms & signs, RDC/TMD AEI: best-fit line 1-way ANOVA, Logistic regression analysis, *p* < 0.05	Case: Unilateral DDWR and arthralgia (n = 12) Bilateral DDWR and no arthralgia (n = 12) Control: asymptomatic individuals (n = 12)	No significant differences (*p* > 0.05) between groups for AEI	No factors associated with the concomitant presence of arthralgia in patients with DDWR
Rabelo, 2017 [17]	Cohort study	199 joints of 104 patients (86F, 18M) m.a.:40.92 a.r.:18-88	TMDs: with TMD symptom AEI: top-roof line 1-way ANOVA, Mann- Whitney rank-sum test, Tukey post hoc test, *p* < 0.05	Classified by shape of fossa (Flattened n = 45; Sigmoid n = 78; Box n = 57; Deformed n = 19)	AEI was higher in box shaped group	Disc position is not influenced by articular eminence morphology AEI has an influence on disk reduction
				Classified by position of disc (Normal n = 86, Displaced n = 113)	AEI were not related to the presence or absence of DD	
				Classified into 8 groups based on types of DD	AEI were not related to the type of DD and AEI	
				Displaced group (n = 113) divided into 2 subgroups (DDWR n = 85, DDWOR n = 28)	AEI was higher for DDWR group	
Aydin, 2012 [41]	Cohort study	70 joints of 35 selected patients (17F, 18M)	TMDs: signs and symptoms AEI: best-fit line Mann–Whitney U- test	DDWR (n = 51) DDWOR (n = 19) Two groups then subdivided by distributions of AEI (shallow (15°–30°), moderate (30°–60°), steep (60°–90°))	No correlation between the 2 groups and AEI (*p* >0.05) For the distributions of AEI in both groups, moderate was the most frequent, followed by shallow and steep	The AEI may not have a predisposing effect on development of DD
Sülün, 2001 [42]	Case-control study	112 joints of 56 symptomatic patients (44F, 12M, m.a.: 33.35) 50 joints of 25 symptom-free volunteers (14F, 11M, m.a.: 23.87)	TMDs: symptoms, confirmed disk malpositions by MRI AEI: best-fit line Mann-Whitney U test, Wilcoxon matched pairs test, *p* < 0.05	Case: DDWR: n = 61, DDWOR: n = 28, Asymptomatic side of the patients: n = 23 Control: AV: n = 50	The AEI on the central and the medial slices in the DDWR group were steeper than those in the AV joints and in DDWOR joints The AEI of the medial slice was larger than the central and lateral slices in DDWR group	A steeper slope is a factor of DD. The flattening observed in the bone surface during the DDWOR stage

M: male; F: female; a.r.: age range; m.a.: mean age; ANOVA: analysis of variance; DD: disk displacement; DDWR: disk displacement with reduction; DDWOR: disk displacement without reduction; AV: asymptomatic volunteer; RDC/TMD: research diagnostic criteria for temporomandibular disorders; AEI: articular eminence inclination.

**Table 4:** Summary of findings from studies of TMDs and AEI with CBCT or Helical CT.

First Author & Year	Type of Study	Population	Intervention	Comparison (Control Group)	Outcome	Conclusions
Al-Rawi, 2017 [43]	Case-control study	70 participants (a.r.: 16–44): 35 TMD patients (19M, 16F, m.a.: 27.9), 35 patients without TMD history (19M, 16F, m.a.: 24.7)	TMDs: RDC/TMD; AEI: top-roof line; Paired sample t-test, independent sample t-test, *p* < 0.05	Case: TMD patients (n = 35); Control: patients without TMD history (n = 35)	AEI was significantly greater in the affected joints in male group, but no difference between affected and normal joints in female group	The condyles of the affected joints may rotate inward
Paknahad, 2016 [14]	Case-control study	40 patients (28F, 12M) with TMD, a.r: 21-57; 23 participants (18F, 5M) without TMD, a.r.: 25-50	TMDs: according to the Helkimo index; AEI: top-roof line; Paired t-test, Student’s t-test, *p* ≤ 0.05	Case: patients with signs and symptoms of TMDs; Control: participants without signs and symptoms of TMDs	AEI was higher in patient group than in control group (*p* = 0.001) No significant difference between the two genders in control group and patient group (*p* > 0.05)	AEI was steeper in patients with TMD
Imanimoghaddam, 2016 [44]	Case-control study	50 patients: 25 TMD patients (5M, 20F, m.a.: 28.84 ± 9.84), 25 normal patients (8M, 17F, m.a.: 28.43 ± 3.24)	TMDs: symptoms & signs, RDC/TMD; AEI: tangent line from the uppermost point of the glenoid fossa; independent t-test, *p* < 0.05	Case: patients suffering from TMD (n = 25); Control: patients with normal TMJs and Class I occlusion (n = 25)	AEI did not differ between the normal and TMD patients	CBCT could be considered a useful diagnostic imaging modality for TMD patients
Shahidi, 2013 [18]	Cohort study	60 joints of 30 patients (21 F, 9M), m.a.: 31.89, a.r: 18-52	TMDs: according to the Helkimo index; AEI: top-roof line; Spearman’s correlation test, paired t test, *p* < 0.05	Classified into 3 groups regarding the clinical Di of the Helkimo index (Di I (n = 5), Di II (n = 18), Di III (n = 7)	No correlation between the 3 groups (Di I, II, and III) and AEI in either joint (*p* > 0.05)	No apparent relationship between the AEI and the clinical Di in patients with TMD
Sümbüllü, 2012 [3]	Case-control study	104 joints of 52 patients (41F, 11M) with TMDs and 82 joints of 41 patients (24F, 17M) without TMDs	TMDs: clinical signs and symptoms; AEI: top-roof line, best-fit line; One-way ANOVA, student’s t-test, *p* < 0.05	Case: patients with TMJ dysfunction; Control: patients without TMJ dysfunction	There was a difference in AEI between the patient and control groups (*p* < 0.05) No differences in AEI according to gender and age in TMD group (*p* > 0.05)	AEI was steeper in healthy control group than in TMDs group
Estomaguio, 2005 [45]	Cohort study	39 female orthodontic patients with TMDs	TMDs: signs and symptoms; AEI: best-fit line; Unpaired t-test, *p* < 0.05	NBC: n = 18, m.a.: 19.1 ± 4.7, a.r.: 15-23; BCBC: n = 21, m.a.: 22.7 ± 7.5, a.r.: 14-30	Lateral and central sections of AEI was steeper in NBC than in BCBC	Flattening of the eminence accompany condylar change
Yamada, 2004 [46]	Cohort study	42 joints of 21 female TMD patients scheduled for orthognathic surgery	TMDs: signs and symptoms; AEI: best-fit line; Mann–Whitney U-test, one- factor ANOVA, *p* < 0.05	Classified by bone change (NBC: n = 20, m.a.: 21.44, a.r.: 17.9-24.6; BCBC: n = 22, m.a.: 22.8, a.r.: 17.5-24.3)	AEI in the lateral and central sections were steeper in NBC group than BCBC group	Flattening of the eminence seems to occur during changes from erosion to osteophyte, and from DDWR to DDWOR.
				BCBC subdivided by types of bone change (erosion: n = 10; osteophyte: n = 12), NBC: n = 20	AEI in all three sections of the osteophyte group were less than in the NBC group	
				Classified by DD (normal: n = 15; displacement: n = 27)	No significant differences between the normal group and displacement group	
				Displacement group then subdivided into 2 groups (DDWR: n = 7; DDWOR: n = 20)	AEI of central and lateral sections in DDWR were steeper than DDWOR	

M: male; F: female; a.r.: age range; m.a.: mean age; ANOVA: analysis of variance; DD: disk displacement; DDWR: disk displacement with reduction; DDWOR: disk displacement without reduction; NBC: no bone change; BCBC: bilateral condylar bone change; AEI: articular eminence inclination.

**Table 5:** Summary of findings from studies of TMDs and AEI with two-dimensional radiographs.

First Author & Year	Type of Study	Population	Intervention	Comparison (Control group)	Outcome	Conclusions
Pullinger, 2002 [19]	Case-control study	162 female patients with unilateral disk disorders (m.a.: 33.68 ± 13.89); 21 asymptomatic female subjects (m.a.: 24.2 ± 2.9)	TMDs: RDC/TMD; AEI: best-fit line; Classification tree analysis, independent samples t test, *p* < 0.05	Case: patients with unilateral disk disorders (DDWR: n = 84; DDWOR: n = 78;) Control: asymptomatic female subjects (n = 21)	No difference in eminence slope angle between 2 groups	
Sato, 1996 [47]	Case-control study	91 joints of 79 females with ADD (m.a.: 24.5 ± 4.90); 48 joints of 24 females without TMDs (m.a.: 21.5 ± 2.45)	TMDs: clinical sign, confirmed with arthrography; AEI: top-roof line, best-fit line; Student’s t-test, *p* < 0.05	Case: joints with ADD (n = 91), then subdivided into DDWR (n = 46) and DDWOR (n = 45); Control: joints without TMJ dysfunction (n = 48)	AEI (best-fit line) of joints with ADD was significantly larger than control joints (*p* < 0.01); No difference of AEI (top-roof line) between the joints with ADD and the control joints; No difference in any variable studied (best-fit line and top-roof line) between DDWR and DDWOR	A steep AEI appears to be partly responsible for the genesis of ADD
Ren, 1995 [20]	Case-control study	34 joints of 34 asymptomatic volunteers (18F, 16M, m.a.: 28, a.r: 18-44); 85 joints of 71 patients (50F, 21M, m.a.: 38, a.r: 21-70)	TMDs: pain in TMJ area, confirmed with arthrography; AEI: best-fit line; ANOVA, paired t-test, *p* < 0.05	Case: ADD joints (n = 85) divided into DDWR (n = 37) and DDWOR (n = 48). Then subdivided by OC: with OC (DDWR = 7, DDWOR = 27), without OC (ADR = 30, ADNR = 21); Control: asymptomatic joints (n = 34)	No difference in the AEI between normal joints and joints with DD in the central and medial sections. (*p* > 0.05). A tendency of a flat eminence in the joints with DDWOR; Normal joints steeper than OC joints in lateral (*p* < 0.01) and medial (*p* < 0.05) section. Joints without OC steeper than Joints with OC (*p* < 0.05)	A steep eminence could not be verified as a predisposing factor for DD; Flattening of the eminence was not related to the DD but OC
Galante, 1995 [48]	Case-control study	74 patients (62F, 12M); 35 asymptomatic volunteers (15F, 14M)	TMDs: with TMD symptom AEI: best-fit line; Chi-square tests, Student-Newman-Keuls tests, *p* < 0.05	Case: patients are classified by MRI into 4 groups (SN, DDWR, DDWOR, DDN/DJD, the simple size of different groups was not mentioned); Control: volunteers are classified by MRI into 2 groups (AV: n = 29, ABN: n = 6)	No difference among the six diagnostic groups.	AEI may not represent a predisposing factor for the development of internal derangement of the TMJ
Panmekiate, 1991 [49]	Cohort study	60 joint of 54 patients	TMDs: Disk position classified by arthrography; AEI: top-roof line; Two-tailed t test, *p* < 0.05.	Superior disc position (20 joints from 17 patients, m.a.: 38); DDWR (20 joints from 19 patients, m.a.: 32); DDWOR (20 joints from 18 patients, m.a.: 33)	No differences in angulation among 3 section in each 3 group	No correlation between a steep articular eminence and ADD.

M: male; F: female; a.r.: age range; m.a.: mean age; ANOVA: analysis of variance; RDC/TMD: research diagnostic criteria for temporomandibular disorders; DDWR: disk displacement with reduction; DDWOR: disk displacement without reduction; ADD: anterior disk displacement; AEI: articular eminence inclination; DD: disk displacement; OC: osseous changes; DDN/DJD: disk displacement without reduction with degenerative joint disease; TMJ: temporomandibular joint.

**Table 6:** Studies retrieved in full text and excluded from the review.

Positive			Negative		
First Author & Year	Radiological Method	NOS Score	First Author & Year	Radiological Method	NOS Score
Rabelo, 2017 [17]	MRI	4	Poluha,2020 [40]	MRI	6
Al-Rawi, 2017 [43]	CBCT	4	Imanimoghaddam M, 2016 [42]	CBCT	5
Paknahad, 2016 [14]	CBCT	5	Shahidi, 2013 [18]	CBCT	5
Sümbüllü, 2012 [3]	CBCT	5	Aydin, 2012 [41]	MRI	4
Estomaguio, 2005 [44]	Helical CT	4	Pullinger, 2002 [19]	2D (tomograms)	5
Yamada, 2004 [45]	Helical CT	3	Ren, 1995 [20]	2D (tomograms)	4
Sülün, 2001 [46]	MRI	4	Galante, 1995 [48]	2D (laminagraph)	2
Sato, 1996 [47]	2D (transcranial radiographs)	3	Panmekiate, 1991 [49]	2D (tomograms)	3

NOS score, Newcastle-Ottawa Scale, three categories with a score of level of evidence ranging from 0 to 9 points to determine the quality of case-control and cohort studies.

## Data Availability

No new data were created or analyzed in this study. Data sharing is not applicable to this article.
